# Trust and all-cause mortality: a multilevel study of US General Social Survey data (1978–2010)

**DOI:** 10.1136/jech-2018-211250

**Published:** 2018-10-15

**Authors:** Giuseppe Nicola Giordano, Jan Mewes, Alexander Miething

**Affiliations:** 1 Department of Clinical Sciences, Genetic and Molecular Epidemiology Unit, Lund University, Malmö, Sweden; 2 Department of Sociology, Lund University, Lund, Sweden; 3 Department of Public Health Sciences, Stockholm University, Stockholm, Sweden

**Keywords:** mortality, multilevel modelling, social capital, social epidemiology, public health

## Abstract

**Background:**

Within public health research, generalised trust has been considered an independent predictor of morbidity and mortality for over two decades. However, there are no population-based studies that have scrutinised both contextual-level and individual-level effects of generalised trust on all-cause mortality. We, therefore, aim to investigate such associations by using pooled nationally representative US General Social Survey (GSS) data linked to the National Death Register (NDI).

**Methods:**

The combined GSS–NDI data from the USA have 90 contextual units. Our sample consisted of 25 270 respondents from 1972 to 2010, with 6424 recorded deaths by 2014. We used multilevel parametric Weibull survival models reporting HRs and 95% CI (credible intervals for Bayesian analysis). Individual-level and contextual-level generalised trust were the exposures of interest; covariates included age, race, gender, marital status, education and household income.

**Results:**

We found a robust, significant impact of individual-level and contextual-level trust on mortality (HR=0.92, 95% CI 0.88 to 0.97; and HR=0.96, 95% CI 0.93 to 0.98, respectively). There were no discernible gender differences. Neither did we observe any significant cross-level interactions.

**Conclusion:**

High levels of individual and contextual generalised trust protect against mortality, even after considering numerous individual and aggregated socioeconomic conditions. Its robustness at both levels hints at the importance of psychosocial mechanisms, as well as a trustworthy environment. Declining trust levels across the USA should be of concern; decision makers should consider direct and indirect effects of policy on trust with the view to halting this decline.

## Introduction

Individual-level determinants of morbidity and mortality have long been recognised as behavioural,^1^ biological^2^ and their possible interaction.^3^ Overarching these are those contextual-level health determinants, which also affect individuals’ morbidity and mortality. These include environmental determinants (eg, access to healthcare resources and reduced exposure to air pollutants),^4^ alongside less tangible contextual phenomena, such as social integration and cohesion.^5^ It is argued that effective political and social institutions help create effective government, which in turn provides favourable conditions for a flourishing civil society, with greater social cohesion, high generalised trust and better health.^5 6^

Generalised trust is an abstract attitude that conceptualises the belief that most people, including strangers, can be trusted. It is considered analytically and conceptually distinct from ‘particularised’ trust, that is, trust in known individuals/groups,^7^ and ‘political trust’, that is, trust in institutions.^6^ The foundations of generalised trust are still often debated. Some consider it an unstable attribute, it being the sum of experiences (good or bad) at any given time.^8^ Others consider that generalised trust is nurtured during one’s formative years, it being stable over the life couse.^7 9^

That generalised trust and health are positively associated is nothing new. From the field of public health, a plethora of empirical ‘social capital’ research has shown support for the hypothesis that generalised trust (at the *individual level and aggregate level*) is an independent health determinant.^10^ However, the relevance of ‘social capital’ has been contested by proponents who highlight the greater importance of public welfare policy and access to material resources for health outcomes.^11^ Furthermore, there has been debate surrounding the suitability of trust as a social capital proxy.^12 13^ Despite this, the vast majority of trust and health research comes under the ‘social capital’ umbrella, with associations between morbidity and *generalised* trust persisting, even in complex analyses.^14^

Potential mechanisms to how generalised trust equates to better health *at the contextual level* include that more trusting/cohesive communities maintain greater access to local health services and amenities, reduce ‘deviant health-related behavior’ and levels of violent crime and facilitate the rapid dissemination of positive health messages and behaviours throughout the collective.^15^ *At the individual level*, trust is considered to positively influence health by lowering everyday ‘transaction costs’, that is, high generalised trust facilitates collective action, reciprocity and social reinforcement.^16^ Routinely low transaction costs, therefore, imply reduced psychosocial stresses and anxiety.^17^ Conversely, low trust/high transaction costs increase social stress and anxiety, which may lead to long-term elevation of blood cortisol levels (due to overstimulation of the hypothalamic-pituitary-adrenal (HPA) axis). Chronically high blood cortisol levels are associated with an increased risk of deleterious diseases, such as type 2 diabetes and cardiovascular disease.^17^

Empirical evidence of associations between generalised trust and *mortality* seems sparse and inconsistent in comparison. A recent review by Choi *et al* 18 found just two studies that employed generalised trust, concluding that there was no significant correlation. Furthermore, a prospective US study concluded that associations initially reported by Kawachi *et al* 19 between aggregated trust, income inequality and mortality were most likely confounded by ethnicity; however, Deaton and Lubotsky did not include any trust measures themselves.^20^ Conversely, another US study showed that higher neighbourhood trust was associated with lower neighbourhood total mortality rates after adjusting for socioeconomic status and ethnicity.^21^ Finally, a cross-national examination of 19 Organisation for Economic Co-operation and Development countries further cast doubt on previously reported positive associations between trust and life expectancy.^22^

Studies employing *individual*-level trust and mortality seem restricted to findings from two Nordic countries. First, from Finland, a study of individuals aged 30–79 years found that the negative association between trust and mortality vanished after adjusting for social participation.^23^ A second study from Southern Finland established an association between men’s (but not women’s) trust and mortality.^24^ Conversely, a prospective study from Northern Denmark found that trust predicted mortality in women only.^25^

Of further interest here are those recent discussions around a possible genetic component of generalised trust thought to be shared by specific (inherited) personality traits.^26^ From the field of health psychology, distrust is the key feature of a character trait known as ‘cynical hostility’.^27^ Individuals who have cynical, mistrusting outlooks also have a more unhealthy psychosocial risk profile^16 17^ and a greater risk of mortality.^27 28^ In trust and mortality research, therefore, a multilevel approach is required to distinguish empirically between individuals who distrust people as part of their pathological personality trait from those who perceive their environment as untrustworthy. Debate surrounding the suitability of trust in social capital research aside,^12^ no study of trust and all-cause mortality has attempted to disentangle associations between individuals’ generalised trust/distrust and aggregate-level trust (*contextual trustworthiness - social cohesion*) with general population data. We, therefore, aim to address this shortfall by using a nationally representative sample from US General Social Survey (GSS) data (1978–2010), combined with the National Death Index (NDI) until 2014.

## Methods

### Data

#### Population

We drew survey data from the combined GSS–NDI database.^29^ The GSS started as a nationally representative, full-probability sample of adults aged 18 years and over in the USA in 1972. Data collection was conducted annually until 1994 and biennially thereafter. Face-to-face interviews with one adult per household were held in English and, from 2006, in Spanish also. Response rates were high, ranging from 70% (2000) to 80% (1987).^29^ The matched GSS–NDI dataset includes records for 12 558 validated deaths through to 2014, linked to GSS data from 1978 to 2010. We removed cases with missing values on generalised trust, education, marital status, income and age. The final working sample (1978–2010) consisted of 25 270 respondents, clustered by region and size of place of residence. Of those, there was a validated death record for 6424 participants by 2014.

#### Event

The studied event was ‘time to death’, with observation time in years as timescale. We right-censored and excluded respondents older than 89 years. Of those respondents who had died by 2014, approximately 54% were from the survey years 1978–1988, 33% were interviewed between 1989 and 1999 and 13% were participants between 2000 and 2010.

#### Multilevel structure

Our hierarchical models distinguished individuals (*level 1*) and contextual units (*level 2*). Legal restrictions meant we could not use ‘federal states’ as *level 2* units of aggregation. To obtain an appropriate number of contextual units, we grouped nine larger regions (US Census Divisions) by the size of respondents’ place of residence. The US Census Divisions comprises ‘New England’, ‘Middle Atlantic’, ‘East North Central’, ‘West North Central’, ‘South Atlantic’, ‘East South Central’, ‘West South Central’, ‘Mountain’ and ‘Pacific’. Our variable ‘size of place’ included 10 categories: (1) city >250 000; (2) city 50 000–250 000; (3) suburb, large city; (4) suburb, medium city; (5) unincorporated, large city; (6) unincorporated, medium city; (7) city, 10 000–49 999; (8) town >2500; (9) smaller areas; and (10) open country. The combinations of both variables (*9*×*10*) resulted in 90 context-level units. Online supplementary appendix table A1 provides a descriptive overview of those 90 level 2 units.

10.1136/jech-2018-211250.supp1 Supplementary file 1

#### Explanatory variable

Generalised trust was measured through the question: ‘Generally speaking, would you say that most people can be trusted, or that you cannot be too careful in dealing with other people?’. Response categories were ‘most people can be trusted’ (‘trust’), ‘can’t be too careful’ (‘distrust’) and ‘it depends’ (as standard, the latter two were recoded as ‘distrust’).^30^ Overall, distrusters (62%) outnumbered trusters (38%), with trust declining from 43% in the 1980s to 34% in the 2000s. To overcome collinearity problems, we centred individual trust scores around the respective cluster-specific, level 2 group mean (mean=0; min=−0.61; max=0.82).

#### Covariates

We considered the following variables potential confounders: age, gender, race (black, white and other), degree (less than high school, high school, junior college, bachelor and graduate), marital status (married, widowed, separated, divorced and never married) and household income (measured in 10 000s of constant 1986 US$, adjusted for household size and centred around the cluster-specific group mean; min=−3.65; max=12.88, SD=1.87).

#### Contextual variables

Contextual trust was aggregated from the individual trust measures and z-standardised (mean=0; min=−2.43; max=2.85; SD=1). Similarly, we z-standardised aggregated household income (mean=0, min=−2.55; max=4.24; SD=1). Using Stata’s user-defined programme INEQDECO,^31^ we computed cluster-specific values concerning income inequality, measured by the Gini coefficient (z-standardised, mean=0; min=−3.13; max=2.51; SD=1).

#### Statistical analyses

We used a parametric proportional hazard model with mixed-effects and Weibull distribution, reporting HRs and 95% CIs in the subsequent analyses. As a robustness check, the fully adjusted model was additionally run within a Bayesian framework (Markov Chain Monte Carlo (MCMC)). Regions, in combination with size of place of residence, were used as second-level units. To overcome potential collinearity, we further ran two separate models (fully adjusted) with either level 1 trust or level 2 trust (see supplementary appendix table A2). All analyses were performed in Stata (V.15).^32^ The GSS probability weight WTSSALL was employed throughout except in our robustness check (as weighting is not allowed in models with the Bayes prefix in Stata).

## Results

Table 1 shows generalised trust stratified by our covariates. Approximately one-third of respondents (37%) trusted others, with ‘trusters’ predominantly being white, educated, married and materially affluent survey participants.

**Table 1:** Generalised trust, stratified by covariates

	Share of trusters (percentage)	n
*Overall*	37.82	25 270
*Sex*		
Female	35.87	13 855
Male	40.18	11 415
*Race*		
Black	15.41	3226
White	42.26	20 601
Other	24.39	1443
*Degree*		
Less than high school	21.98	4340
High school	35.08	13 348
Junior college	37.21	1634
Bachelor	53.42	4073
Graduate	60.53	1875
*Marital status*		
Married	42.34	13 355
Widowed	36.74	1704
Divorced	35.22	3663
Separated	24.12	912
Never married	31.32	5636
*Household income (measured as a continuous variable in multilevel models)*		
Lowest quartile *(poorest)*	23.93	6096
2nd quartile	33.31	6295
3rd quartile	42.28	6363
Highest quartile *(richest)*	50.80	6516

Source: GSS–NDI (1978–2010).

n, 25 270;

NDI, National Death Index; GSS, General Social Survey.

Contextually, the South stands out as an environment with particularly low social cohesion (table 2). Only 29% of respondents in West South Central and 27% of respondents in East South Central agree with the statement that most people can be trusted. Conversely, about 49% of inhabitants in West North Central tended to trust other people. A more detailed look at the distribution of trust across the 90 contextual (*level 2*) variables (see online supplementary appendix table A1) shows that, on average, people living in big cities are less trusting than those living in the suburbs and in smaller sized communities.

**Table 2:** Distribution of trust across US Census Divisions

US Census Division	Share of trusters (percentage)	n
New England*	46.75	1183
Middle Atlantic†	37.67	3401
South Atlantic‡	32.51	4804
East North Central§	41.02	4620
East South Central¶	27.03	1728
West North Central**	48.52	1923
West South Central††	28.85	2399
Mountain‡‡	44.33	1737
Pacific§§	40.37	3475

The US Census Division units comprise the following US states:

*New England: Connecticut, Maine, Massachusetts, New Hampshire, Rhode Island and Vermont.

†Middle Atlantic: New Jersey, New York and Pennsylvania.

‡South Atlantic: Delaware, District of Columbia, Florida, Georgia, Maryland, North Carolina, South Carolina, Virginia and West Virginia.

§East North Central: Indiana, Illinois, Michigan, Ohio and Wisconsin.

¶East South Central: Alabama, Kentucky, Mississippi and Tennessee.

**West North Central: Iowa, Nebraska, Kansas, North Dakota, Minnesota, South Dakota and Missouri.

††West South Central: Arkansas, Louisiana, Oklahoma and Texas.

‡‡Mountain: Arizona, Colorado, Idaho, New Mexico, Montana, Utah, Nevada and Wyoming.

§§Pacific: Alaska, California, Hawaii, Oregon and Washington,

Data source: GSS–NDI (1978–2010). n=25 270.

NDI, National Death Index; GSS, General Social Survey.

Model 1 (table 3) tested the association between level 2/level 1 trust (centred around grand/group means), controlling for age. We found support for an association between trust and mortality, with trusting respondents having a 17% lower risk of dying over the observation time than their distrusting counterparts (HR=0.83, 95% CI 0.79 to 0.87). Irrespective of individual trust levels, respondents from high-trust contexts had lower mortality than those from low-trust regions (and vice versa). We further ran a modified version of model 1 including a random coefficient for level 1 trust and a cross-level interaction between level 2 and level 1 trust (results not shown). However, no significant interaction was observed, nor any evidence for meaningful variation in level 1 trust.

**Table 3:** Determinants of all-cause mortality: results from multilevel parametric Weibull proportional hazard regression models (HRs and 95% CIs)

	Model 1		Model 2	
	HR	95% CI	HR	95% CI
*Individual level*				
Generalised trust (group-mean centred)	**0.83**	*0.79 to 0.87*	**0.92**	*0.88 to 0.97*
Age	**1.07**	*1.07 to 1.08*	**1.07**	*1.07 to 1.08*
*Race*				
Black (ref: white)			**1.24**	*1.13 to 1.36*
Other race			1.03	*0.91 to 1.16*
*Sex*				
Female (ref: male)			**0.69**	*0.65 to 0.73*
*Degree*				
Less than high school High school (ref:)			**1.21**	*1.13 to 1.29*
Junior college			**0.84**	*0.73 to 0.97*
Bachelor			**0.84**	*0.77 to 0.91*
Graduate			**0.87**	*0.78 to 0.97*
*Marital status*				
Married (ref.)				
Widowed			**1.22**	*1.14 to 1.31*
Divorced			0.99	*0.92 to 1.08*
Separated			1.11	*0.97 to 1.26*
Never married			**1.32**	*1.21 to 1.44*
*Income*				
Household income (in 10 000 constant 1986 US$, group-mean centred)			**0.94**	*0.93 to 0.96*
*Contextual level*				
Generalised trust (level 2, aggregated level 1 trust, z-standardised)	**0.93**	0.90 to 0.96	**0.96**	*0.93 to 0.99*
Ln(*p*)		0.504		0.512
Residual variance (level 1)		0.600		0.593
Residual variance (level 2)		0.006		0.004
Level 2 variance explained		60%		73%

Residual variance, level 1=0.921; residual variance, level 2**=**0.015

Data source: GSS–NDI: n=25 270; number of failures=6424. Data weighted with WTSSALL.

Statistically significant coefficients (p<0.05) boldfaced.

NDI, National Death Index; GSS, General Social Survey.

Model 2 (table 3) considered level 1 and level 2 trust, age, sex, race, education, marital status and household income as confounders. The statistically significant association between mortality and level 1 and level 2 trust remained (HR=0.92, 95% CI 0.88 to 0.97; and HR=0.96, 95% CI 0.93 to 0.99, respectively). Again, we did not find support for the presence of a cross-level interaction between level 2 trust and level 1 trust in a modified version of model 2 (*not shown*). An intraclass coefficient of 0.016 (1.6%) was derived from the partitioned variance of the empty model. Model 2 (table 3) accounted for 73% of this level 2 variation. We further tested an interaction between respondents’ gender and generalised trust, finding no evidence for an effect modification of the trust-mortality association by gender. Neither did we find significant interaction effects between trust and any of our covariates (results not shown).

In table 4 (model 3.1), we used the same covariates as model 2, while also controlling for the contextual units’ mean income and their income inequality to test if associations between contextual trust and mortality still held. Mirroring results elsewhere,^19^ level 2 trust demonstrated a strong correlation with income inequality and a modest correlation with mean income. When adjusting for level 2 income inequality and mean income, the model still yielded a statistically significant HR regarding level 2 trust. Given its borderline significance (z-value=1.96), we ran model 3.2 within a Bayesian framework (table 4). Using MCMC estimation with a 15 000 burn-in and a chain of approximately 270 000 iterations (model acceptance rate=0.33, efficiency <0.03 for all model parameters; effective sample size >200 for all coefficients), we found further evidence for a robust association between contextual trust and mortality (HR=0.96; 95% credible interval 0.94–0.98). From our separate level 1 and level 2 trust models (see supplementary appendix table A2), level 1 trust had a stronger association with individual mortality than level 2 trust. However, both are relatively similar in terms of effect size.

**Table 4:** Determinants of all-cause mortality: results from multilevel parametric frequentist versus Bayesian Weibull proportional hazard regression models (HRs with 95% CI and credible interval, respectively)

	Model 3.1		Model 3.2	
	*Frequentist*		*Bayesian*	
	HR	95% CI	HR	95% CI
*Individual level*				
Generalised trust (group-mean centred)	**0.92**	*0.88 to 0.97*	**0.94**	*0.91 to 0.98*
Age	**1.07**	*1.07 to 1.08*	**1.07**	*1.07 to 1.08*
*Race*				
Black (ref: white)	**1.24**	*1.13 to 1.36*	**1.20**	*1.13 to 1.28*
Other race	1.03	*0.91 to 1.16*	1.05	*0.97 to 1.13*
*Sex*				
Female (ref: male)	**0.69**	*0.65 to 0.73*	**0.69**	*0.67 to 0.72*
*Degree*				
Less than high school High school (ref:)	**1.21**	*1.13 to 1.29*	**1.21**	*1.17 to 1.26*
Junior college	**0.84**	*0.73 to 0.97*	**0.89**	*0.81 to 0.97*
Bachelor	**0.84**	*0.77 to 0.91*	**0.89**	*0.85 to 0.92*
Graduate	**0.87**	*0.78 to 0.97*	**0.82**	*0.77 to 0.88*
*Marital status*				
Married (ref.)				
Widowed	**1.22**	*1.14 to 1.31*	**1.23**	*1.15 to 1.32*
Divorced	0.99	*0.92 to 1.08*	0.99	*0.95 to 1.04*
Separated	1.05	*0.97 to 1.26*	1.11	*1.00 to 1.10*
Never married	**1.32**	*1.21 to 1.44*	**1.26**	*1.21 to 1.33*
*Income*				
Household income (group-mean centred)	**0.94**	*0.93 to 0.96*	**0.94**	*0.93 to 0.95*
*Contextual level*				
Generalised trust (z-standardised)	**0.96**	0.93 to 0.99*	**0.96**	*0.94 to 0.98*
Income inequality (GINI-coefficient, z-standardised)	0.99	*0.96 to 1.03*	1.00	*0.98 to 1.02*
Mean income (z-standardised)	0.99	*0.95 to 1.02*	0.99	*0.96 to 1.01*
Ln(*p*)		0.512		
Residual variance (level 1)		0.593		
Residual variance (level 2)		0.004		
Level 2 variance explained		73%		

Notes: statistically significant coefficients (p<0.05) boldfaced. Residual variance, level 1=0.921, residual variance, level 2= 0.015. Data source: GSS–NDI: n=25 270; number of failures=6424. Data weighted with WTSSALL (model 3.1).

*Full CI interval*: 0.9252366- 0.999961.*

NDI, National Death Index; GSS, General Social Survey.

## Discussion

The aim of this study was to investigate associations between individual-level and aggregate-level generalised trust and all-cause mortality in the USA. Using pooled (1978–2010) US GSS data (N_T_=25 270) merged with National Death Index data (events=6424 by 2014) and applying multilevel parametric Weibull survival regression, we found a significant negative association between generalised trust (*at both levels)* and mortality that held in a fully adjusted Bayesian MCMC model (see table 4, model 3.2). Furthermore, and contradicting previous findings from Denmark and Finland, we found no evidence of any effect modification by gender.^23–25^

Key hypotheses emerging from literature that attempt to explain associations between high trust and longevity are briefly discussed below.

### Trust and ‘social capital’

From social and political sciences, trust is often considered an important pillar of modern social capital theory.^33 34^ Distinct from particularised trust, however, generalised trust is favoured when considering the social cohesion perspective, it being frequently used to capture environmental ‘trustworthiness’ at both the contextual *and* the individual level.^10 16 35^

If trust is considered a valid social capital proxy,^13^ then those (contextual level and individual level) mechanisms from trust to health previously mentioned provide plausible explanations for its positive effects on health outcomes. However, recent research suggests that trust may well be conceptually distinct from social capital,^12^ supporting the idea that high trust may capture flourishing civil society and effective government, contexts in which populations are likely to thrive.^5 6^

### Cynical hostility and ‘distrust’

Originating from the field of health psychology, measures of ‘distrust’ are core components to hostility scale constructs.^16^ From these, levels of cognitive hostility (*expressed*/*externalised*), adverse hostility (*internalised*), anger and aggression can be quantified.^36^ Interestingly, high levels of adverse/internalised hostility have been shown to predict mortality, even after adjusting for socioeconomic and adverse health behaviours.^28^

The (biologically) plausible pathways from high levels of cynical hostility to mortality most likely involve the psychosocial/HPA axis,^17^ which are well documented in health psychology and psychosomatic medicine literature.^16^

At an individual level, it is impossible to distinguish distrust (a pathology) from individual perceptions of environmental cohesiveness. Our multilevel approach, however, shows that both individual and aggregate trust robustly protect against mortality.

Levels of generalised trust have been in decline across the USA since the 1960s, attributed by some to lower levels of social participation.^34^ Others have argued that lower US trust levels are a response to increasing income inequality.^37^ High degrees of social inequality have been linked to the erosion of social cohesion in society and higher levels of distrust.^19^ The perception of social inequalities and (dis)trust may also affect individuals’ ability to mobilise social support,^38^ high levels of which have also been associated with better health. Trusters may have greater access to these kinds of resources, enabling them to cope better with any social disadvantage and/or potential psychosocial health hazard(s).^17^

Perhaps now is the time for US decision makers to use those benefits of egalitarian policies. Though such policies are designed specifically to reduce inequalities (eg, in wealth, health and opportunity), they may also reduce individual *perceptions* of inequality, thus further nurturing a more ‘trusting’ milieu. Interestingly, a recent publication investigating the impact of the Affordable Care Act on health and trust provides empirical support for this notion.^39^

### Strengths and weaknesses

This is the first study to investigate individual and aggregate trust and all-cause mortality using rich nationally representative US data, spanning more than three decades. It is statistically not possible to differentiate between individuals’ distrust (a pathology) and individual-level perceptions of environmental trustworthiness. However, we employed a multilevel design specifically to disentangle individual-level effects from any contextual/aggregate measures of social cohesion and trustworthiness. The negative association between generalised trust (*at both levels)* and mortality held in our robustness checks; however, these findings should be interpreted with caution, as the GSS probability weights cannot be employed within Stata’s MCMC routines, potentially biasing our findings.

Even though a recent study based on UK panel data showed how individuals’ generalised trust can change,^8^ levels do tend to revert back to an initial (longer term) trust level,^9^ rendering our findings more credible.

While our pooled US GSS data are nationally representative, our study design relied on attitudinal observations from a single point in time. Furthermore, our approach missed capturing how changes in income and marital status could affect mortality; this also holds true for contextual changes regarding income and income inequality.

Finally, legal constraints hindered us from employing US states as contextual units. Future research should replicate our design with these as level 2 units, with their more ‘objective’ data on income and income inequality. Despite this ‘unorthodox’ approach, our contextual clusters had similarly strong correlations between trust, mean income and income inequality, as reported in other studies using state-level data.^19^

## Conclusion

This US-based study demonstrated a clear survival advantage for trusters compared with distrusters, both at individual and aggregate levels. The association between generalised trust and mortality was robust, even after accounting for numerous socioeconomic conditions. The persistent impact of trust on mortality, over and above those conditions of income inequality that contribute to the social gradient in mortality, hints that psychosocial mechanisms are at play.^17^ If higher trust levels are a potential resource to increase individuals’ resilience towards health hazards arising from social disadvantage, then the decline in trust seen across the US over past decades is of concern. Decision makers, therefore, should consider any impact that policies may also have on trust, with the view to halting or even reversing this decline.

**What is already known on this subject**

- From the field of public health, individual and aggregated generalised trust (eg, at neighbourhood, community and state levels) are associated with morbidity and mortality.
- The field of psychology, however, also considers individual-level ‘distrust’ as a predictor of mortality.
- Currently, there is no multilevel study employing individual mortality data alongside contextual and individual-level trust that could disentangle such associations.

**What this study adds**

- Distrusting US Americans have a higher risk of death than those who trust others. Declining trust levels across the USA should be of concern. Decision makers should consider direct and indirect effects of policy on trust with the view to halting this negative trend.
