# Supplementary material for: Trust and all-cause mortality: a multilevel study of US General Social Survey data (1978–2010)
Source: J Epidemiol Community Health. 2018 Oct 15;73(1):50–5. doi: 10.1136/jech-2018-211250 (PMC6839792; doi:10.1136/jech-2018-211250)
Supplement: Supplementary file 1 [file jech-2018-211250supp001.pdf]

TableA1: The distribution of trust across the 90 contextual (*level 2*) variables

	NORC ext size	n	Region number	Trust (mean)	Income in real 1986 USD, adjusted for hh size (mean)	Income inequality (Gini)
Overall				.38	21124.79	.43
New England	City gt 250k	56	1	.39	25171.81	.52
New England	City, 50k-250k	253	2	.44	22415.56	.42
New England	Suburb, lrg city	126	3	.45	29600.23	.39
New England	Suburb, med city	287	4	.47	26049.11	.41
New England	Uninc, lrg city	44	5	.39	40089.59	.38
New England	Uninc, med city	180	6	.55	32502.31	.33
New England	City, 10k-49,999	24	7	.50	17751.09	.31
New England	Town gt 2500	98	8	.44	19914.30	.38
New England	Smaller areas	53	9	.45	17679.70	.33
New England	Open country	62	10	.52	21449.55	.44
Middle Atlantic	City gt 250k	794	11	.27	19995.77	.50
Middle Atlantic	City, 50k-250k	274	12	.27	15567.16	.45
Middle Atlantic	Suburb, lrg city	960	13	.40	25948.35	.41
Middle Atlantic	Suburb, med city	341	14	.40	22378.06	.40
Middle Atlantic	Uninc, lrg city	350	15	.48	27730.78	.37
Middle Atlantic	Uninc, med city	257	16	.47	26548.46	.38
Middle Atlantic	City, 10k-49,999	77	17	.47	18892.08	.36
Middle Atlantic	Town gt 2500	93	18	.39	14095.10	.42
Middle Atlantic	Smaller areas	91	19	.45	15119.80	.37
Middle Atlantic	Open country	164	20	.44	19856.67	.40
E. Nor. Central	City gt 250k	744	21	.30	20206.65	.47

E. Nor. Central	City, 50k-250k	618	22	.42	18643.08	.43
E. Nor. Central	Suburb, lrg city	1003	23	.42	25954.27	.41
E. Nor. Central	Suburb, med city	402	24	.44	25273.94	.39
E. Nor. Central	Uninc, lrg city	206	25	.43	23076.56	.40
E. Nor. Central	Uninc, med city	475	26	.49	20211.26	.43
E. Nor. Central	City, 10k-49,999	271	27	.38	16485.01	.41
E. Nor. Central	Town gt 2500	242	28	.42	16932.26	.39
E. Nor. Central	Smaller areas	223	29	.52	16804.66	.36
E. Nor. Central	Open country	436	30	.38	15622.99	.38
W. Nor. Central	City gt 250k	218	31	.39	16283.00	.52
W. Nor. Central	City, 50k-250k	280	32	.45	22468.99	.40
W. Nor. Central	Suburb, lrg city	318	33	.49	24463.98	.37
W. Nor. Central	Suburb, med city	122	34	.48	21983.35	.37
W. Nor. Central	Uninc, lrg city	85	35	.53	23555.05	.36
W. Nor. Central	Uninc, med city	49	36	.55	28902.82	.36
W. Nor. Central	City, 10k-49,999	215	37	.48	16368.16	.40
W. Nor. Central	Town gt 2500	147	38	.57	16232.50	.37
W. Nor. Central	Smaller areas	290	39	.54	16595.63	.36
W. Nor. Central	Open country	199	40	.45	17336.58	.47
South Atlantic	City gt 250k	484	41	.34	21854.67	.43
South Atlantic	City, 50k-250k	677	42	.33	20909.49	.46
South Atlantic	Suburb, lrg city	772	43	.36	24653.73	.41
South Atlantic	Suburb, med city	701	44	.29	21114.46	.44
South Atlantic	Uninc, lrg city	457	45	.40	31780.21	.42
South Atlantic	Uninc, med city	413	46	.32	18451.83	.38
South Atlantic	City, 10k-49,999	388	47	.32	15923.52	.45
South Atlantic	Town gt 2500	254	48	.35	19534.36	.45
South Atlantic	Smaller areas	149	49	.29	16725.18	.43
South Atlantic	Open country	509	50	.24	14141.42	.43

Supplementary File /Appendix

E. South. Central	City gt 250k	185	51	.37	21270.93	.48
E. South. Central	City, 50k-250k	243	52	.24	14021.46	.41
E. South. Central	Suburb, lrg city	191	53	.32	20903.49	.40
E. South. Central	Suburb, med city	116	54	.27	15286.41	.36
E. South. Central	Uninc, lrg city	47	55	.28	18210.02	.34
E. South. Central	Uninc, med city	204	56	.29	19200.26	.43
E. South. Central	City, 10k-49,999	152	57	.18	11730.37	.45
E. South. Central	Town gt 2500	80	58	.21	12585.62	.44
E. South. Central	Smaller areas	281	59	.26	13199.45	.47
E. South. Central	Open country	229	60	.26	16923.92	.44
W. South Central	City gt 250k	562	61	.34	21401.29	.44
W. South Central	City, 50k-250k	212	62	.22	14275.55	.48
W. South Central	Suburb, lrg city	410	63	.35	21887.31	.42
W. South Central	Suburb, med city	202	64	.29	13612.39	.42
W. South Central	Uninc, lrg city	135	65	.28	26359.64	.49
W. South Central	Uninc, med city	46	66	.30	19768.71	.46
W. South Central	City, 10k-49,999	236	67	.27	18247.74	.44
W. South Central	Town gt 2500	188	68	.20	17338.94	.48
W. South Central	Smaller areas	119	69	.32	14781.78	.44
W. South Central	Open country	289	70	.22	13490.95	.48
Mountain	City gt 250k	338	71	.37	13490.95	.47
Mountain	City, 50k-250k	365	72	.47	21868.70	.43
Mountain	Suburb, lrg city	217	73	.48	23610.45	.52
Mountain	Suburb, med city	123	74	.43	18737.25	.50
Mountain	Uninc, lrg city	31	75	.61	18836.37	.33
Mountain	Uninc, med city	78	76	.46	22702.04	.42
Mountain	City, 10k-49,999	286	77	.42	19041.24	.39
Mountain	Town gt 2500	167	78	.44	14807.67	.46

Mountain	Smaller areas	28	79	.36	9725.057	.40
Mountain	Open country	104	80	.54	17109.65	.42
Pacific	City gt 250k	708	81	.39	22776.85	.47
Pacific	City, 50k-250k	580	82	.38	21190.10	.47
Pacific	Suburb, lrg city	1240	83	.40	27901.38	.43
Pacific	Suburb, med city	420	84	.38	21763.07	.43
Pacific	Uninc, lrg city	53	85	.57	22514.10	.38
Pacific	Uninc, med city	92	86	.49	25992.00	.37
Pacific	City, 10k-49,999	134	87	.41	19411.75	.43
Pacific	Town gt 2500	97	88	.42	20483.11	.37
Pacific	Smaller areas	73	89	.53	21093.30	.37
Pacific	Open country	78	90	.51	21093.30	.41

Table A2: Associations between individual-level trust (model A1) vs. contextual-level trust (model 2) and individual mortality (Hazard ratios (HR) and 95% confidence intervals)

	Model A1		Model A2	
	HR	95% CI	HR	95% CI
Individual level				
Social trust (Ref: distrust)	**0.92**	*0.87-0.97*		
Age	**1.07**	*1.07-1.08*	**1.07**	*1.07-1.08*
Race				
Black (Ref.: white)	**1.26**	*1.15-1.38*	**1.26**	*1.15-1.38*
Other race	1.03	*0.91-1.16*	1.04	*0.92-1.17*
Sex				
Female (Ref.: male)	**0.69**	*0.65-0.73*	**0.69**	*0.65-0.73*
Degree				
Less than high school	**1.22**	*1.14-1.30*	**1.22**	*1.15-1.30*
High school (ref.:)				
Junior college	**0.84**	*0.73-0.97*	**0.84**	*0.73-0.97*
Bachelor	**0.83**	*0.77-0.91*	**0.83**	*0.76-0.90*
Graduate	**0.87**	*0.77-0.97*	**0.85**	*0.76-0.95*
Marital status				
Married (ref.)				
Widowed	**1.23**	*1.14-1.31*	**1.23**	*1.15-1.31*
Divorced	0.99	*0.92-1.08*	1.00	*0.92-1.08*
Separated	1.11	*0.97-1.26*	1.11	*0.98-1.27*
Never married	**1.32**	*1.21-1.44*	**1.32**	*1.21-1.44*
Income				
Household income in 10,000 USD (group-mean centred)	**0.94**	*0.93-0.96*	**0.94**	*0.92-0.96*
Contextual level				
Generalised trust (z-standardized)			**0.96**	*0.93-0.99*
Ln(*p*)		.511		.488
Residual variance (level 2)		.005		.003
